# 

**Published:** 1885-09

**Authors:** 


### Ohio State Dental Society—Annual Meeting.

The annual meeting of this society will be held in Chillicothe, on Wednesday, the 29th of October. An excellent programme has been prepared by the Executive Committee, and will be sent out to the profession in a few days. A reorganization was effected last year, and it is very desirable that the good men of the profession in the State take hold and do what they can for the upbuilding and strengthening of the society. The following are the officers and committes:

President, C. H. James, Cincinnati; Vice-President, H. H. Harrison, Cadiz; Secretary, J. R. Callahan, Hillsboro; Treasurer, Geo. W. Keely, Oxford.

Standing Committees: Executive—F. H. Rehwinkel, Chillicothe; C. R. Butler, Cleveland; E. G. Betty, Cincinnati. Revision of State Law—F. H. Rehwinkel, Chillicothe; F. A. Hunter, Cincinnati; C. H. James, Cincinnati. Voluntary Essays—J. Taft, Cincinnati; Geo. Watt, Xenia; D. Gibbons, Warren. Publication—W. H. Sillito, Xenia; J. R. Callahan, Hillsboro; H. H. Harrison, Cadiz. State Board of Examiners—H. A. Smith, C. R. Butler, I. Williams, J. Taft, F. H. Rehwinkel. Membership, C. H. Harrison, Toledo; A. F. Emminger, Columbus; G. W. Keely, Oxford. Ethics—F. H. Rehwinkel, Chillicothe; C. H. James, Cincinnati; I. Williams, New Philadelphia.

### CONTENTS.

### COMMUNICATIONS.

Anæsthetics ... 429
Treatment of Pulpless Teeth ... 433
The Management of Children in the Dental Chair ... 443
The Use of Cocaine in Dentistry ... 448
The Treatment of Deep-seated Abscesses without External Incision 453
Anæsthesia ... 458

### PROCEEDINGS.

Report ... 468
SALMAGUNDI ... 472

### EDITORIAL.

American Dental Association ... 477

Ohio State Dental Society—Annual Meeting ... 480

---

### THE DENTAL REGISTER,

A MONTHLY MAGAZINE DEVOTED TO THE INTERESTS OF THE

**DENTAL PROFESSION.**

*Terms Reduced to* $2.00 *Per Year. Single Copies,* 25 *Cents.*

**EDITED BY PROF. J. TAFT, D.D.S.**

—AND—

**Published by SAM'L A. CROCKER & CO., Ohio Dental and Surgical Depot.**

The volume commences in January and closes in December.

The REGISTER being issued monthly is always fresh, therefore Indispensable to the practitioner who desires to keep posted up with the new inventions and improvements which are daily developed. Neither expense nor effort will be spared to give value and variety to its contents. Articles will be illustrated with well executed engravings whenever they will add to the interest or assist to explanation.

Its extensive circulation and frequency of issue make it one of the BEST DENTAL ADVERTISING MEDIUMS in the United States.

All subscriptions must begin with January or July.

Local or special notices, five lines or less, will be inserted for $3 each insertion; over five lines, 50 cts. per line.

All advertising bills payable in advance, or at furthest, after the issue of the first number containing the advertisement. All transient advertisements to be paid for in advance.

☞CONTRIBUTIONS SOLICITED.

Exclusively Editorial Communications should be addressed to the Editor.

The latest number of the REGISTER may be ordered with or without other goods at any time, and all letters should be addressed to

**SAM'L A. CROCKER & CO., Ohio Dental and Surgical Depot, Cincinnati, O.**

